# POTENTIAL BENEFITS OF THC AND CBD IN THE TREATMENT OF HOSPICE-ELIGIBLE DEMENTED AND AGITATED PATIENTS

**DOI:** 10.1093/geroni/igac059.396

**Published:** 2022-12-20

**Authors:** Jacobo` Mintzer

**Affiliations:** University of South Carolina, Columbia, South Carolina, United States

## Abstract

Today, half of the patients suffering from Alzheimer’s disease (AD) will use hospice care in the last days of their life. Most of them will present with moderate to severe agitation. In the absence of evidence-based guidelines, Hospice care eligible patients with Agitation and Alzheimer’s disease (AD) or other types of Dementia (HAD) are treated with a combination of antipsychotics, benzodiazepines, and opiates, which generate a variety of side effects. Two cannabinoids, tetrahydrocannabinol (THC) and cannabidiol (CBD), appear to be promising therapies for agitation in HAD with minimal side effects. Specifically, we suggest that a combination of THC and CBD oils have enhanced synergistic effects while maintaining a low side effect profile that the combination may provide. The evidence for this hypothesis will be discussed during the symposium.

